# tRNA diversification among uncultured archeon clones

**DOI:** 10.6026/97320630014357

**Published:** 2018-07-31

**Authors:** Mohammad Mahfuz Ali Khan Shawan, Md. Ashraful Hasan, Raihana Yesmin, Tareq Hossan, Md. Mozammel Hossain, Md. Mahmudul Hasan, Afroza Parvin, Mahbubul Morshed, Nahiyan Mohammad Salauddin, Satya Ranjan Sarker, Md. Nazibur Rahman, S. M. Badier Rahman

**Affiliations:** 1 Department of Biochemistry and Molecular Biology, Jahangirnagar University, Savar, Dhaka-1342, Bangladesh; 2 Department of Soil, Water and Environment, University of Dhaka, Bangladesh; 3 Department of Biotechnology and Genetic Engineering, Jahangirnagar University, Savar, Dhaka-1342, Bangladesh

**Keywords:** Uncultured archeon clones, true/functional tRNA, NCBI BioSample database, GC percentage, tRNAscan-SE 2.0 tool, ENDMEMO GC calculating tool, tRNA type, cove score

## Abstract

Whole genome sequences (DNA sequences) of four uncultured archeon clones (1B6:CR626858.1, 4B7:CR626856.1, 22i07:JQ768096.1 and 19c08:JQ768095.1) were collected from NCBI BioSample database for the construction of digital data on tRNA. tRNAscan-SE 2.0 and ENDMEMO tools were used to identify and sketch tRNA structure as well as calculate Guanine-Cytosine (GC) percentage respectively. Eight true/functional tRNAs were identified from above 4 sequences which showed cove score greater than 20% with no variable loop. The tRNAs from the uncultured archeon clones were classified as Ala, Arg, Ile, Thr, Pro and Val type tRNA with cove score ranging from 34.22%-79.03%. The range of GC content was found 42.89%-56.91%; while tRNA contributed GC content ranging from 52%-64.86% to the total GC content in these sequences. The data fabricated in this study could be very useful for studying the diversity of tRNA among prokaryotes.

## Background

Transfer RNA (tRNA), a tiny non-coding RNA comprises of about 75-95 nucleotides (nts). It is ubiquitous in all the three domains of life and concerned with translation machinery in deciphering mRNA to protein [1]. A secondary structure made up of a terminal helical stem and three hairpin loops is the distinguishing features of all tRNAs. The functional parts of a tRNA include the anticodon triplets that interpret the mRNA codons and the 3' CCA nts that is charged with corresponding amino acid delivering into the ribosome during translation [2]. The highly complex classes of genes within tRNA are still evolving and the analysis of tRNA diversity is an exhilarating topic in the field of molecular evolution [3]. Diversification of these ancient macromolecules (tRNA) seems to be co-evolved with RNA splicing endonucleases under string evolutionary pressure to which diverse genetic lineages were adapted in translation. Mitochondrial oxidative environment also probably had the influence on tRNA evolution [4]. Archeal genome reveals three types of tRNA genes namely non-intronic tRNA (encoded on a single gene with no intron), intronic tRNA (encoded on a single gene with 1-3 introns) and split tRNA (found only in hyperthermophilic archeal parasite and encoded on separate genes). The evolutionary study of tRNA genes clarifies that ancestral tRNA was encoded on a single gene or separate genes [3]. Thus, understanding of diverse true/functional tRNA fragments will help us to detect the systematic classification of fragments in the context of full-length tRNA genes. The knowledge on evolved tRNA will also guide us to solve the different quests such as whether it is evolved from common ancestor, or whether it is lost during evolution.

### Dataset

Whole genome sequences of four uncultured archeon clones (CR626858.1, CR626856.1, JQ768096.1 and JQ708095.1) were downloaded in FASTA format through NCBI's BioSample database. Data on tRNA was detected and scrutinized through tRNAscan-SE 2.0 tool. Perceived tRNAs were categorized into different types on the basis of coded amino acid and cove score. ENDMEMO GC content calculator was used to generate data on GC content in percentage.

### Experimental design, materials and methods

Complete DNA sequences of four uncultured archeon clones were retrieved from NCBI (National Center for Biotechnology Information) BioSample database via Nucleotide DNA database and stored in FASTA format [5]. Detected tRNAs were classified into different classes based on amino acid code and cove score [6, 7, 8, 9]. ENDMEMO GC calculating tool was used to generate the data on GC percentage for both the whole genome sequences and detected tRNAs [10]. ENDMEMO GC plotting tool was utilized to illustrate pattern of GC allocation through graphical representations. Within the GC plot, upper and lower red lines specify highest and lowest percentage of GC allotment, while middle blue line demonstrates average GC percentage distributed in DNA sequence [11, 12, 13, 14].

## Results & discussion

**Figure 1:**
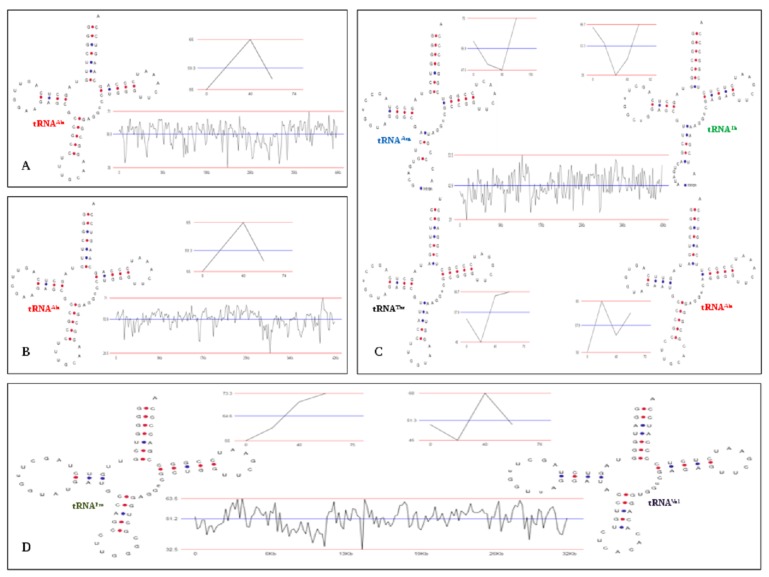
tRNA secondary structure and GC allocation through graphical representations in four uncultured archeon clones detected by tRNAscan-SE 2.0 and ENDMEMO GC calculator. A) 1B6:CR626858.1; B) 4B7:CR626856.1; C) 22i07:JQ768096.1 and D) 19c08:JQ768095.1

In this study, detection, classification as well as function and structure prediction of tRNA genes within four uncultured archeon clones were achieved by using newly developed tRNAscan-SE 2.0 tool, which has advanced state of art methodology in tRNA gene investigation and uses genomic tRNA database having rich new content [15]. This online tool classifies tRNA into different types depending on amino acid code and cove score [6]. As shown in Table 1, this investigation identified single Ala type tRNA (tRNAAla) having cove score of 78.59% with no introns for each of CR626858.1 and CR626856.1. Additionally, this tool detected four tRNAs (tRNAAla, tRNAArg, tRNAIle and tRNAThr) having cove score ranging from 34.22%- 79.03% with two introns (tRNAArg and tRNAIle), and two tRNAs (tRNAPro and tRNAVal) having cove score ranging from 64.70%- 74.24% with no introns for JQ768096.1 and JQ768095.1 respectively. Thus, it is evident that, all of these tRNAs have cove score more than 20% and can be considered as true/functional tRNA. Furthermore, six out of eight predicted tRNAs having no introns imply non-intronic while rest of the two predicted tRNAs with introns indicate intronic tRNAs. Previously, Rekadwad et al. using complete genome sequences of two uncultured archaea and ten uncultured bacteria observed a total of seven archaeal tRNAs (tRNAAla, tRNAArg and tRNACys) having cove score ranging from 54.34%-75.97% and fourty eight bacterial tRNAs (tRNAAla, tRNACys, tRNAGln, tRNAGlu, tRNAIle, tRNALeu, tRNALys, tRNAMet, tRNAPhe, tRNAPro, tRNASer and tRNAVal) having cove score ranging from 58.09%-97.15%. In both cases, no introns within the tRNAs were obtained [9]. Interestingly, no selenocysteine tRNAs (TCA), suppressor tRNAs (CTA and TTA), pseudogenes and tRNAs with unknown isotypes were found in both the present investigation and the study carried out by Rekadwad et al. [9]. Determination of GC content within the whole genome as well as tRNA is very much crucial, because extremely high or low level of genomic GC content may produce an unassigned codon by losing a tRNA [16]. As shown in Figure 1, GC allocation through graphical representations reveal approximately 56.9%, 53.9%, 42.9% and 51.2% of GC in the 1B6:CR626858.1, 4B7:CR626856.1, 22i07:JQ768096.1 and 19c08:JQ768095.1 respectively, with tRNAs having GC ranging from 51.3%-64.6%. This finding is consistent with the observation of Rekadwad et al. who also found GC content approximately 43% for archaeal genome, wherein archaeal tRNA contributed 60.4%-64.2% GC to the total GC content [9].

## Conclusion

This study identifies and analyzes true/functional tRNAs using whole genome sequences (complete DNA sequences) that has spawned novel data on true tRNA diversity among the four uncultured archeon clones. Data on GC content and digitization of these novel tRNAs appear to be white snow for research on tRNA and made available to users.

## Conflict of interest

The authors do not declare any competing interest.

## Figures and Tables

**Table 1:** Results for the analysis of true/functional tRNA detected in four uncultured archeon clones using tRNAScan-SE 2.0

Sequence name	No. of tRNA	tRNA begins	Bounds end	tRNA type	Anti-codon / at	Intron begins	Bounds end	Cove score (%)	tRNA length (bp)
1B6:CR626858.1	1	30548	30476	Ala	TGC/30515-30513	0	0	78.59	73
4B7:CR626856.1	1	33664	33592	Ala	TGC/33631-33629	0	0	78.59	73
22i07:JQ768096.1		12593	12741	Arg	ACG/12626-12628	12629	12705	34.22	149
		25074	25164	Ile	GAT/25109-25111	25113	25128	67.36	91
		26821	26894	Thr	GGT/26855-26857	0	0	74.14	74
	4	24399	24328	Ala	TGC/24367-24365	0	0	79.03	72
19c08:JQ768095.1		19803	19730	Pro	TGG/19769-19767	0	0	64.7	74
	2	2177	2103	Val	CAC/2142-2140	0	0	74.24	75
